# The Molecular and Functional Interaction Between Membrane-Bound Organelles and Membrane-Less Condensates

**DOI:** 10.3389/fcell.2022.896305

**Published:** 2022-04-25

**Authors:** Chuankai Zhou

**Affiliations:** ^1^ Buck Institute for Research on Aging, Novato, CA, United States; ^2^ Leonard Davis School of Gerontology, University of Southern California, Los Angeles, CA, United States

**Keywords:** membrane-bound organelles, membrane-less condensates, stress granules, protein aggregates, phase separation

## Abstract

A major recent advance in cell biology is the mechanistic and kinetic understanding of biogenesis of many membrane-less condensates. As membrane-less condensates and membrane-bound organelles are two major approaches used by the eukaryotic cells to organize cellular contents, it is not surprising that these membrane-less condensates interact with the membrane-bound organelles and are dynamically regulated by the cellular signaling, metabolic states, and proteostasis network. In this review, I will discuss recent progress in the biogenesis of membrane-less condensates and their connections with well-studied membrane-bound organelles. Future work will reveal the molecular and functional connectome among different condensates and membrane-bound organelles.

## Introduction

One major goal of cell biology is to understand the principles and mechanistic details behind the self-organization of cellular contents into individual functional units of different scales. For example, most cell biologists study the biogenesis and functions of membrane-bound organelles in eukaryotic cells, which spatiotemporally and dynamically compartmentalize unique parts of proteome, nucleic acids, lipids, and metabolites to efficiently and specifically carry out different cellular functions. The studies on each individual organelles are now gradually continued by the efforts to elucidate the interaction among different membrane-bound organelles, mainly motivated by the intention to better understand cellular physiology as most cellular functions are fulfilled by multiple steps across different organelles. Although the prototypical organelles are membrane-bound, membrane-less cellular compartments (now often called condensates) have been observed since the very beginning of cell biology research (Montgomery, 1898; Wilson, 1899). The recent years have seen an explosive interest in these membrane-less condensates in the light of their biogenesis through liquid-liquid phase separation (LLPS). With the maturation of the theories behind the biogenesis of membrane-less condensates, it is of great interest to study the inter-connectome of these cellular structures, with and without membrane, to fully understand how the contents and information exchange between them to achieve certain cellular functions. Here I review the biogenesis mechanisms of the membrane-less condensates and their known interactions with membrane-bound organelles.

## The Biogenesis of Membrane-Less Condensates

Our interest on the membrane-less condensates dates back to the discovery of nucleolus (Montgomery, 1898; Wilson, 1899) and centrosomes (Boveri, 1888). This also makes nucleolus and centrosome the most studied condensates. While nucleolus and centrosome represent the common condensates found in almost all eukaryotic cells, other membrane-less condensates are usually found in specific cell types, developmental stages, or induced by certain stresses. For example, post-synaptic density is found in the neurons (Zeng et al., 2016), paraspeckles are found in some epithelial cells (Nakagawa et al., 2011), germline P granules are formed during *C. elegans* embryonic development (Brangwynne et al., 2009), and stress granules (SG) are induced by various stress conditions (Collier and Schlesinger, 1986; Anderson and Kedersha, 2008; Gwon et al., 2021). Except for a few cases, such as the centrosome and post-synaptic density, most of the condensates consist of both proteins and RNA.

The biogenesis of various condensates was initially studied separately to understand the key components and the protein-protein/protein-RNA interactions inside. For example, the formation of SGs was proposed to be nucleated by specific protein-mRNA interaction that forms oligomers which are crosslinked by PABP-1 into microscopically visible SGs (Anderson and Kedersha, 2008). Similarly, a number of nuclear bodies, including nucleolus, histone locus bodies (HLBs), Cajal body, Nuclear splicing speckles, paraspeckle, and nuclear stress bodies (nSB) were nucleated by specific RNAs which recruit additional proteins to form microscopically visible granules (Mao et al., 2011; Shevtsov and Dundr, 2011; Falahati et al., 2016; Falahati and Wieschaus, 2017; Erhardt and Stoecklin, 2020). However, most of these condensates contain hundreds of proteins that their recruitment and interactions remain uncharacterized. For example, nucleolus selectively enrich >700 different nuclear proteins *via* unknown mechanisms.

The recent resurgence of interest on LLPS provides a fresh perspective on the selective enrichment of different components in a membrane-less condensate. Inspired by the examples such as the P granule and nucleolus with liquid properties (Brangwynne et al., 2011, 2009), a surge of publications revisited different membrane-less condensates and propose that LLPS drives the selective condensation and enrichment of different proteins and mRNAs into each membrane-less compartment. LLPS arises from the supersaturation of molecules: given a specific condition, a molecule in a solution will partition into two separate high-concentration and low-concentration phases when its concentration rises above the critical concentration (Banani et al., 2017). It has been shown that multivalent interactions, either from multidomain proteins or intrinsically disordered regions (IDRs), drive the condensation of molecules (Banani et al., 2017). In this model, multivalent proteins, or scaffold proteins, crosslink with each other to setup the framework which recruits client proteins with lower valency (Banani et al., 2016). Although classical cases of LLPS driven by multidomain proteins have been reported, such as the Nephrin-Nck-N-WASP (Li et al., 2012), most of the condensation events are made possible by the IDRs-mediated multivalent weak interactions. Indeed, proteomes of different membrane-less condensates are enriched in IDRs, such as RNA-binding proteins, which can search multiple conformations (Crick et al., 2006; Mukhopadhyay et al., 2007; Tran et al., 2008; Darling et al., 2018) and form weak intermolecular interactions through the cation-pi, electrostatic, and polypeptide backbone interactions (Xiang et al., 2015; Hughes et al., 2018). It is important to note that although most studies focused on the IDRs-mediated multivalent protein-protein interactions (PPIs), many condensates are dominated by RNAs (Van Treeck and Parker, 2018; Roden and Gladfelter, 2021). For example, the G3BP1 and RNA in the SG is about 1 mg/ml and 64 mg/ml, respectively (Guillén-Boixet et al., 2020). As mRNAs are at least three times longer than polypeptides, it has been suggested that multivalent RNA-RNA and RNA-protein interactions likely dominate the nucleation and condensation of molecules (Zhang et al., 2015; Langdon et al., 2018; Van Treeck and Parker, 2018; Roden and Gladfelter, 2021). These multivalent RNAs and scaffold proteins provide the attractive model in which different client proteins can be recruited to the membrane-less condensates through non-specific weak interactions and LLPS, thus potentially explain the selective recruitment of hundreds of different proteins.

Although the IDR-based multivalent weak interaction and LLPS now are the default explanations for the condensation of membrane-less compartments, we should note that the traditional specific protein-protein/protein-RNA interactions among folded protein and RNA domains also play critical roles (McSwiggen et al., 2019b). For example, although some of the nucleolar proteins can automatically associate and condense into microscopically visible structure, their spatiotemporal localization are nucleated by specific protein-RNA interactions (Falahati et al., 2016). Additionally, some nucleolar proteins are recruited through active process instead of thermodynamic LLPS (Falahati and Wieschaus, 2017). Thus, for a membrane-less condensate that shows phase separation behavior for some of its components, there are many proteins that are recruited *via* alternative mechanisms. In another case, live-cell single-molecule imaging revealed that transcription factors (TFs) form hubs *via* multivalent interactions between IDRs without showing LLPS, which happens only when the TFs are overexpressed (Boija et al., 2018; Chong et al., 2018). A recent study on Herpes Simplex Virus replication compartment (RC) showed that although RC displays hallmarks of LLPS, including roundness, fission and fusion, and speedy fluorescence recovery, single particle tracking suggested that RC is formed through non-specific protein-DNA interaction without forming two different phases (McSwiggen et al., 2019a).

**FIGURE 1:**
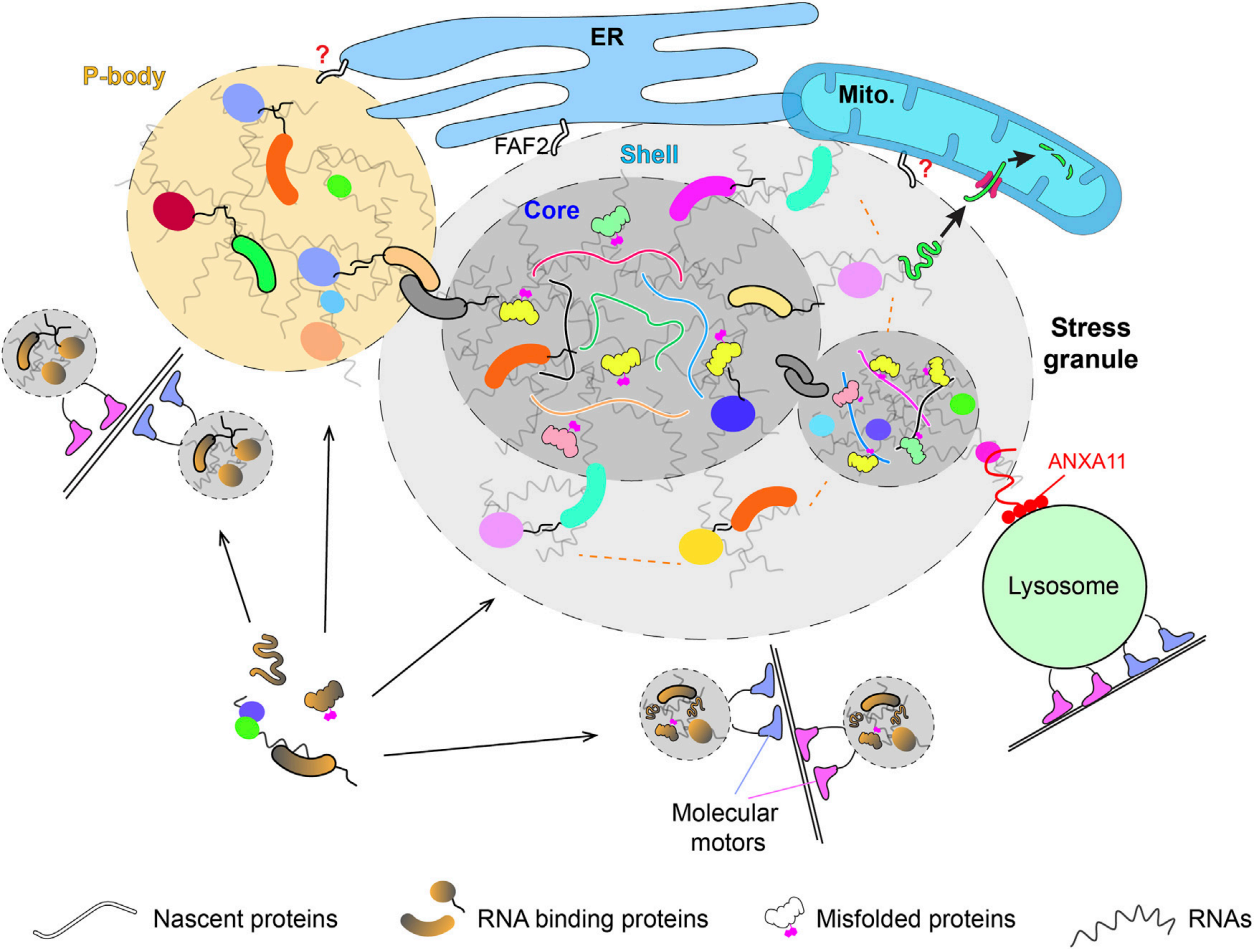
The biogenesis and interaction of membrane-less condensates with organelles. P-body and stress granule interact with membrane-bound organelles in different organisms. The spatially localized biogenesis of these condensates suggests certain key membrane-bound molecules initiate their assembly. The recruitment of different components to the condensates happens *via* both LLPS and alternative mechanisms, such as the active transport by molecular motors. The abundant RNAs in these condensates likely dominate the multivalent weak interactions required to assemble and stabilize these membrane-less structures with help from IDP-containing scaffold proteins. The heterogeneity of endogenously assembled condensates is reflected by the anisotropic properties within each individual condensate (e.g., shell and core in SG) and the heterogenous molecular composition across different condensates in the same cell. The recruitment of nascent and misfolded proteins into the core of SG likely solidifies the structure. The condensate-membrane interaction allows some misfolded proteins to get imported into and degraded inside the mitochondria or hitchhike on lysosomes for long-distance transport.

The contribution of both thermodynamic LLPS and other alternative mechanisms to the formation of membrane-less condensates are probably best illustrated by the heterogeneity within the condensates formed *in vivo*, while LLPS alone predicts largely homogenous constitution throughout the condensate. Super resolution studies showed that SGs, P granules, paraspeckles, and RCs have anisotropic properties across the compartment (West et al., 2016; Jain et al., 2016; Wheeler et al., 2016; McSwiggen et al., 2019a; Wang et al., 2014). In the case of SGs, the condensates are composed of stable cores surrounded by a phase separated shell (Jain et al., 2016) (Figure 1). Although such stable cores can be explained by the aging of liquid condensates following LLPS (Molliex et al., 2015; Patel et al., 2015; Xiang et al., 2015), evidence showed that weak nonspecific interactions underlying LLPS are not required for the formation of stable cores, and importantly, the size of these stable cores does not change overtime (Wheeler et al., 2016). In addition, early results showed that the formation of microscopically visible SGs relies on multiple cellular factors, including dynein and kinesin (Loschi et al., 2009; Kwon et al., 2007). The dependence of SG formation on these motors contradicts to the LLPS-aging model and instead, is consistent with a model in which the stable cores of SGs are nucleated through active process into which additional factors are recruited *via* LLPS (Figure 1). This LLPS independent mechanism seems not unique to SG, as the isolation of endogenously formed p-bodies (PB) and nucleoli into protein-free buffers does not cause the dissolution of these condensates as predicted by LLPS and previously showed with *in vitro* reconstituted condensates (Hubstenberger et al., 2017; Hayes et al., 2018). Consistently, while LLPS predicts a dynamic exchange of components between different phases, quantification of different PB components revealed that some of the key components show little to no exchange with the surrounding cytosol (Xing et al., 2020). Similarly, the LLPS-mediated condensations of endocytic factors and ZO proteins are initiated by scaffold proteins (Syp1 or tight junction receptors) vis a LLPS-independent process (Beutel et al., 2019; Bergeron-Sandoval et al., 2021).

## The Interactions Between Membrane-Less Condensates and Membrane-Bound Organelles

Many membrane-less condensates are spatially close to or interact with each other, such as paraspeckles vs. nuclear speckles and PB vs. SG (Kedersha et al., 2005; Kedersha and Anderson, 2007; Fox and Lamond, 2010; Sanders et al., 2020). The mechanisms behind the physical interactions of different condensates are currently under extensive exploration, with recent results highlighting the contribution of overlapping PPI networks between different condensates to their physical connectivity (Sanders et al., 2020). There are also cases where the membrane-less condensate binds to the membrane-bound organelles. For example, the P granules tightly associate with the nuclear membrane and the nuclear pore complex in the germ cells of *C. elegans* (Pitt et al., 2000). A TIS11B-enriched protein-RNA condensate (TIS granule) interacts with endoplasmic reticulum (ER) (Ma and Mayr, 2018). In addition, the intercellular junctions, such as tight junctions between epithelium cells and nephrin between podocytes, form plasma membrane associated protein condensates that recruit downstream effectors (Banjade and Rosen, 2014; Beutel et al., 2019; Case et al., 2019). A recent paper reported the liquid phase separation behind the recruitment of multiple components of the endocytic coat on the plasma membrane which drive the deformation and internalization of plasma membrane during endocytosis (Bergeron-Sandoval et al., 2021).

PB and SG are archetypal membrane-less condensates used to study the interaction between membrane-less and membrane-bound structures (Figure 1). Early studies in yeast revealed that PB tend to dock on ER (Kilchert et al., 2010). Proteomics study of the PB interacting proteins discovered two ER-associated proteins (Scp160 and Bfr1) that known to interact with polysome (Weidner et al., 2014). However, Scp160 and Bfr1 are not required for the localization of PB to the ER. PB was also found to dynamically associate with ER in mammalian cells (Lee et al., 2020). This recent study showed that the translational capacity on the cisternal ER sheets correlates with the amount of PB and their ER association. As PB are storage sites of dormant mRNAs, it was speculated that the contact between PB and ER allows mRNA to shuffle between repressive and active translation status (Lee et al., 2020). However, PBs did not tend to associate with the cisternal ER where the mRNA is abundant and stripping mRNA/polysomes from ER by puromycin did not detach PB from ER. As the transcriptome inside PB does not enrich mRNAs related to ER or secretory pathway (Hubstenberger et al., 2017), it remains unclear regarding why and how PB interact with ER.

SGs also interact with membrane-bound organelles. Early studies in yeast showed that SGs, or protein aggregates/Q-bodies, that induced by different stresses are associated with ER and mitochondria (Escusa-Toret et al., 2013; Zhou et al., 2014; Böckler et al., 2017). Recent studies in mammalian cells showed that SGs also associate with membrane-bound organelles, such as lysosomes and ER (Lee et al., 2020; Liao et al., 2019; Gwon et al., 2021). The molecular identities of SG-ER/mitochondria/lysosome interaction remain largely unknown. In the case of SG-lysosome interaction, proteomics study identified ANXA11 as a molecular tether that can dynamically couple SGs with lysosomes (Liao et al., 2019) (Figure 1). Although the SGs were shown to have limited interaction with ER in one study (Liao et al., 2019), a separate study showed that SGs are tightly tethered by ER *via* FAF2 which marks the fission of SGs (Lee et al., 2020; Gwon et al., 2021) (Figure 1). The fission events of SGs are rare compared to their fusion events, which dominating the LLPS and liquid condensates, highlighting the heterogeneity of SGs *in vivo* that differ in both compositions and physical properties (Khong et al., 2017). Similarly, PBs also show heterogeneity *in vivo* with individual PB recruits mRNA independently (Wang et al., 2018) and interacts with ER with different dynamics (Lee et al., 2020). These heterogeneities further support the model that these membrane-less condensates are assembled *via* a combination of LLPS and alternative mechanisms (Figure 1).

## The Functions of Condensates-Organelles Interaction

Many molecular and cellular functions have been proposed for different condensates, such as regulating biochemical reactions (Su et al., 2016; Du and Chen, 2018), sequestration of molecules (Frottin et al., 2019; Youn et al., 2019), compartmentalizing/vectorizing the complex modification of molecules (Riback et al., 2020), and buffering stochastic cellular noises (Klosin et al., 2020). It is important to note that due to the multivalent nature of phase separating molecules, most of the studies used extensive mutations/truncations to remove multivalent interacting sites on key scaffold proteins to block their phase separation. These large-scale mutations likely have pleiotropic effects on other functions of the scaffold protein, which is usually a hub in the network of specific PPIs with hundreds of other molecules in addition to its IDR-mediated weak interactions required for LLPS (Hubstenberger et al., 2017; Sanders et al., 2020; Yang et al., 2020). Furthermore, there are examples that the formation of membrane-less condensates is not required for the related functions. For example, removing NEAT-1, the scaffold of paraspeckles, has no effect in the cells and tissues (Nakagawa et al., 2011). Blocking SG formation did not affect the stress-induced translation repression (Kedersha et al., 2016), and the dissolution of SG is not required to restore translation during recovery (Loschi et al., 2009). Furthermore, long-term exposure to the same stressors causes cellular adaptation that prevents the formation of SGs (Domnauer et al., 2021). Similarly, formation of PB is not required for mRNA decay (Decker et al., 2007). Although multiphase nucleolus is proposed to vectorize the assembly of ribosomes in eukaryotes (Riback et al., 2020), there is no such multiphase structure in prokaryotes for ribosome biogenesis. Similarly, previous studies reported a mitochondrial “RNA granule” that recruits several accessory proteins to assemble mitochondrial ribosome (Barrientos, 2015). Instead of LLPS *via* IDR-mediated weak multivalent interactions, recent study showed that these accessory proteins fold and co-assemble with ribosome intermediate (Cheng et al., 2021). Future studies are required to address the complexity of native condensates and the discrepancy among different studies (Lyon et al., 2021).

The interactions between membrane-less condensates and membrane-bound organelles play important roles in the functions and fates of condensates. For example, the ER-associated TIS granules enrich AU-rich mRNAs and enable the interaction between the nascent membrane proteins translated inside TIS granule and SET, which sorts the nascent proteins to different subcellular localizations along the secretory pathway (Ma and Mayr, 2018). The plasma membrane-associated protein clusters increase the dwelling time of proteins inside, enabling kinetic proofreading that enhances the activities of the recruited proteins, such as the Nephrin-Nck-N-WASP for actin polymerization and LAT-Grb2-SOS for Ras activation (Case et al., 2019; Huang et al., 2019). It is important to note that membrane-association is not strictly required for both cases as condensates alone without membrane association can also activate actin assembly or Ras signaling (Li et al., 2012; Tulpule et al., 2021). In the case of ER-PB/SG association, ER tubules wrap the condensates and induce the fission of the ER-associated PB/SG (Lee et al., 2020). The lysosome-SG/RNPs interaction mediates the long-range transportation of SGs (Liao et al., 2019). This is similar to early studies in yeast where the tight-association between protein aggregates/SG and mitochondria dominate the motility of these SGs (Zhou et al., 2014; Böckler et al., 2017). It was shown that most of the SGs induced by heat shock are formed directly on the surface of mitochondria (Zhou et al., 2014), suggesting a spatially organized biogenesis of membrane-less condensates on organelles. In contrast to the long-range active transportation of the lysosome-associated SGs in mammalian cells, the mitochondrial association of SGs in yeast reduces the long-range transportation and contributes to the asymmetric retention of these SGs during mitosis (Zhou et al., 2014).

In addition to the motility of SGs, the association between SGs and mitochondria also contributes to the dissolution of SGs. It was shown in both yeast and mammalian cells that many aggregated cytosolic proteins inside SGs were solubilized by chaperones and imported into mitochondria for their degradation (Ruan et al., 2017; Li et al., 2019; Shcherbakov et al., 2019) (Figure 1). Although mitochondrial import is a selective process under physiological and heathy conditions, it is known that some neurodegenerative diseases related proteins get into mitochondria and cause mitochondrial defects (Devi et al., 2006, 2008; Hansson Petersen et al., 2008; Wang et al., 2016). The mitochondrial import of aggregated non-mitochondrial proteins indicates that misfolded proteins can hijack the mitochondrial import pathway if they are presented in the vicinity of the import channels *via* the mitochondria associated SGs.

It remains largely unclear how and why SGs establish connections with specific membrane-bound organelles. In the case of mitochondria-SG interactions, most of the SGs are formed on the surface of mitochondria and thus maintain their association with mitochondria (Zhou et al., 2014). As the misfolded proteins and RNAs are ubiquitously distributed in the cytosol, this membrane-associated biogenesis of SGs indicates certain spatially localized factors drives the formation of SGs, which resembles the localized nucleation of nucleolus (Falahati et al., 2016). Future studies are required to understand the *in vivo* biogenesis and interaction of SGs and other membrane-less condensates with organelles. It is also critical to elucidate how the biogenesis of membrane-bound organelles are regulated in a way to prevent the aggregation (formation of SGs) of organellar proteins which are aggregation-prone (Wang and Chen, 2015; Wrobel et al., 2015; Liu et al., 2022).

## Conclusion

It is expected that more condensates will be described in the coming years and their interactions with membrane-bound organelles will be a spotlight of future research. Elucidation of the molecular mechanisms linking membrane-less condensates to membrane-bound organelles is critical to understand the function of such interactions. Additionally, future studies will shed light on the fate of different condensates (e.g., asymmetric segregation or degradation *via* autophagy) and their connections with the inter-organellar contact sites (King et al., 2020). In the end, we will understand the evolutionary perspective of the interactions between condensates and organelles, two major ways of organizing the cellular contents.
